# Hypothermia in the operating theatre

**DOI:** 10.1186/cc11275

**Published:** 2012-06-07

**Authors:** Andreas Gruber, Wilhelm Behringer, Engelbert Knosp

**Affiliations:** 1 Department of Neurosurgery, Medical University of Vienna, Austria; 2 Department of Emergency Medicine, Medical University of Vienna, Austria

## Background

When addressing neuroprotective effects and possible indications of therapeutic hypothermia, several scenarios have to be distinguished. First, therapeutic hypothermia can be classified based on the depth of cooling from normal body temperature into mild hypothermia (32 to 35°C), moderate hypothermia (28 to 32°C), deep hypothermia (28 to 17°C), and profound (<17°C). Second, therapeutic hypothermia can be initiated prior to the insult, for preservation of tissue during the insult, or for the reduction of reperfusion injury after an insult. Third, animal studies and clinical trials have addressed the neuroprotective effects of therapeutic hypothermia under conditions of both global and focal ischaemia. Forth, additional parameters, for example, rewarming rate, duration of ischaemia, and extent of reperfusion, influence the effects of therapeutic hypothermia. Fifth, different outcome measures have been used to describe the effects of hypothermia, for example, infarct size, extent of cellular death, and neurologic condition at different time intervals after ischemia. Therefore, whenever discussing procedures involving therapeutic hypothermia, the aforementioned parameters deserve clarification.

## Cerebral ischaemia

Cerebral ischaemia results from a reduction or complete loss of cerebral blood flow (CBF) and lack of cerebral oxygenation, followed by depletion of ATP, dysfunction of ATP-dependent membrane pumps and subsequently occurrence of anoxic depolarisation. A large amount of glutamate is released from the intracellular space into the extracellular space, causing excitotoxic injury by stimulating *N*-methyl-D-aspartate (NMDA) receptors and triggering calcium influx. Increased intracellular calcium levels *per se *amplify injury by increasing calcium permeability and glutamate release via second messenger mechanisms. These acute cascades lead to necrotic neuronal death by interfering with the mitochondrial respiratory chain. Ischaemia and reperfusion further enhance excitotoxicity by providing oxygen as a substrate for several enzymatic oxidation reactions, thereby generating products of reactive oxygen species in large quantities. These free radicals enhance protein oxidation and lipid membrane disintegration and in conjunction with blood-brain barrier (BBB) disruption further contribute to ischaemic necrosis. Apoptosis also occurs in cerebral ischemia, with antiapoptotic proteins being selectively upregulated in surviving neurons and proapoptotic proteins being highly expressed in dying cells.

## Hypothermia in cerebral ischaemia

The first controlled attempts to cool the human brain were undertaken by the neurosurgeon Temple Fay in 1938 [1]. Irrigating the brain directly with ice water and sometimes achieving solid parenchymal freezing, he claimed 'extremely gratifying results' in a paper on 'local and generalized refrigeration of the human brain'. Over time, many mechanisms have been proposed regarding the neuroprotective effect of hypothermia. First, hypothermia results in a temperature-dependent decrease of oxygen and glucose metabolism; that is, a 10°C decrease in temperature reduces ATP consumption and the cerebral metabolic rate (CMR) of oxygen, glucose, and lactate twofold to fourfold [2]. Second, intra-ischaemic hypothermia exerts inhibitory effects on many of the detrimental ischaemic cascades, thereby retarding the initial ATP depletion, preserving metabolic stores, delaying anoxic depolarisation, reducing ischaemia-induced excitotoxic neurotransmitter release and intracellular calcium levels, changing glutamate receptor regulation, and limiting BBB breakdown. Busto and coworkers in 1987 reported that even 1 to 2°C temperature reductions were sufficient to protect against experimental ischemic stroke [3], thereby demonstrating that the aforementioned mechanisms can exceed the effects of temperature-induced reductions in CMR, and in turn providing the pathophysiologic foundation for mild and moderate therapeutic hypothermia in the management of cerebral ischaemia.

## Indications for therapeutic hypothermia

The importance of therapeutic hypothermia has recently been emphasized by randomised trials in patients with global cerebral ischaemia from out-of-hospital cardiac arrest (OHCA) [4] and in neonates with perinatal hypoxic-ischaemic encephalopathy (HIE) [5]. Clinical experience with patients suffering traumatic brain injuries (TBI) and ischaemic strokes are not as convincing, although mild therapeutic hypothermia was sufficient to control intracranial hypertension in this population.

The use of therapeutic hypothermia in today's OR theatres differs significantly from the scenarios outlined in the context of OHCA, HIE, TBI, or stroke. First, in the OR theatre hypothermia is induced beforehand in expectation of a severe cerebral ischaemic challenge caused by the surgical procedure. Second, underexperienced neuro-anaesthesiologist and cardio-anaesthesiologic management, highly invasive procedures including deep hypothermic cardiac arrest (DHCA) and selective cerebral perfusion can be employed. In other words, whereas outside the OR mild to moderate hypothermia is used to treat patients in the post-ischaemic period, deep intra-ischaemic hypothermia can be used in cardiothoracic and neurosurgery for the management of congenital heart disease, thoracic aneurysms, and intracranial aneurysms; that is, interventions provoking global cerebral ischaemia and otherwise resulting in devastating intraoperative strokes. Moreover, mild hypothermia is used during temporary parent artery clipping in cerebral aneurysm surgery, closely resembling a state of mild intra-ischaemic hypothermia in a condition of transient focal cerebral ischaemia.

## DHCA in global cerebral ischaemia

Although DHCA is being used in both cardiothoracic and neurosurgery and usually involves the same principles of extracorporeal circulation under cardiopulmonary bypass (CPB), the rationale behind these procedures is very different.

Hypothermic CPB and DHCA are established strategies of cerebroprotection during cardiothoracic surgery. Cerebral circulatory standstill is an undesired byproduct of this procedure and limits the possible safe duration of surgery. To overcome this problem, methods of retrograde and antegrade cerebral perfusion have been established [6]. Retrograde cerebral perfusion (RCP) was initially considered to extend the safe operative time by both, backward perfusion of the brain via the superior cava vein at pressures of 20 to 30 mmHg, and selective cooling of the brain parenchyma. It has become evident that RCP indeed provides inadequate cerebral perfusion and exerts neuroprotective actions mainly by providing additional cerebral cooling. In contrast, antegrade cerebral perfusion (ACP) is obtained by intermittent infusion of cooled blood directly into cerebral arteries. In detail, selective ACP allows bihemispheric perfusion through direct cannulation of at least two aortic arch vessels, whereas nonselective, hemispheric ACP uses the axillary canula that is used for systemic perfusion as a route for ACP. From the neurosurgical standpoint, not surprisingly, insufficient crossflow across the communicating arteries at the circle of Willis with inadequate perfusion of the contralateral hemisphere has been identified as an important limitation for nonselective ACP. Although a final cooling temperature of 20°C or below with a long cooling time and gradual rewarming are commonly advocated, promising results have been reported by some groups using selective ACP with moderate hypothermia [7].

From the neurosurgical standpoint, the most important information from the cardiothoracic surgical experience is the recommendation that the DHCA time in sole application should not exceed 20 to 25 minutes and in every case with expected DHCA time >25 minutes, ACP or RCP supplement should be performed. In the field of neurosurgery, DHCA combines advanced cerebroprotection with optimal surgical conditions; that is, a blood-free no-flow surgical field and a collapsed aneurysm dome. Since the surgical procedure requires cerebral exsanguination for rapid aneurysm repair, ACP and RCP cannot be employed to dilate the time of DHCA, limiting the safe no-flow period to 20 to 30 minutes.

Management algorithms for intracranial aneurysm surgery under DHCA have been published previously [8-11]. Such advanced cerebrovascular procedures require multimodality neuromonitoring and are performed under barbiturate-induced EEG burst suppression. The decision to use DHCA is usually made intraopertively; that is, hypothermia and cardiac standstill are employed only after microsurgical exploration has proven that safe aneurysm repair is impossible without these adjunctive measures. In this case, brain retractor placement is adjusted, avoiding further retractor repositioning - with the risk of contusional parenchymal haemorrhage - after systemic heparinisation. Thereafter femoro-femoral percutaneous cannulation is performed and after systemic heparinisation (300 to 400 IU/kg) heart-lung extracorporeal circulation with a heat exchanger and oxygenator is started. Once adequate CPB flow is achieved, systemic hypothermia is induced. Cooling during extracorporeal circulation is continued until a desired brain temperature of 14 to 18°C is reached. Hypothermia results in ventricular fibrillation below 28°C and circulatory arrest at 18 to 22°C. At this point CPB is stopped and blood is actively drained into the venous reservoir, rendering the operative field blood free and the aneurysm collapsed. The duration of this circulatory standstill is limited to the duration of surgical aneurysm repair. In the largest reported series [9], the mean duration of circulatory arrest was 21.8 minutes (range 2 to 72 minutes) with a mean temperature during circulatory arrest of 17.2°C (range 12 to 20°C). The decompressed aneurysm can now be dissected circumferentially, adjacent vital perforating branches can be separated from the aneurysm fundus, and in partially thrombosed lesions the sac can be opened and evacuated. Some centres use short but repetitive periods of circulatory standstill followed by periods of reperfusion, thereby reducing the length of a single ischaemic period and checking for bleedings in the operative field [12]. After aneurysm repair, circulation is restored and under slow rewarming the heart starts to fibrillate spontaneously and will either convert to sinus rhythm or will require cardioversion. Pharmacologic peripheral vasodilation is used to facilitate reperfusion, the blood previously drained is reinfused, and heparin is reversed with protamine. Only after reperfusion, rewarming, and reversal of heparin is adequate hemostasis possible in the operative field.

DHCA was used to treat intracranial aneurysms in the 1960s, but shortcomings of CPB and hypothermia-induced coagulopathies resulted in operative morbidity and declining use of the procedure. In the late 1980s, refined microsurgical and anaesthesiologic techniques allowed the use of DHCA for the management of most difficult intracranial aneurysms in selected neurosurgical centres of excellence [8-11]. With the development of new diagnostic and therapeutic strategies, however, DHCA is only rarely needed for the management of cerebral aneurysms today. Possible explanations include the following. First, easy access to improved neuroradiologic imaging techniques has resulted in the detection of cerebral aneurysms at an earlier stage; that is, before they can grow to giant size requiring the aforementioned surgical procedures. Second, endovascular treatment, for example, flow diverter implantation [13] and techniques of flow reversal, has become an alternative to surgery and should in some instances be considered the first-line treatment; for example, complex and giant posterior circulation aneurysms as well as patients with significant co-morbidities. Third, techniques of cerebral revascularisation - that is, parent artery occlusion under bypass protection - have proven highly effective in the management of complex aneurysms [14]. Forth, alternative strategies for intraoperative aneurysm decompression have been discovered. Adenosine-induced cardiac asystole has emerged as a possible alternative, resulting in brief repeated periods of cardiac arrest (5 to 15 seconds) to facilitate aneurysm clipping [15]. Others have used selective brain cooling with extracorporeal femoral to carotid artery perfusion in giant aneurysm surgery [16], a technique differing from DHCA in so far as the patient's body core temperature is maintained at 35°C and aneurysm decompression is achieved by temporary clipping (trapping) of the parent artery under selective brain cooling to 22°C. Another group has reported successful treatment of a giant basilar artery aneurysm using moderate hypothermia and extracorporeal circulation [12].

Even in expert hands, the procedural complication rate of aneurysm surgery under DHCA is significantly higher than in ordinary aneurysm surgery. The largest published series [9] reports a perioperative mortality of 14% and a combined rate of permanent treatment-related morbidity and mortality of 32%. This recent study, however, reported a contemporary population of patients harbouring complex aneurysms not lending themselves to one of the aforementioned alternative treatment forms. In view of the otherwise unfavourable natural course of these aneurysms, the reported 63% good outcome rate justifies the procedure in a small subset of highly selected patients. For the time being, DHCA should be considered a procedure of last resort for otherwise untreatable aneurysms.

## Intra-ischaemic mild hypothermia in focal cerebral ischaemia

The situation of temporary parent artery clipping during cerebral aneurysm surgery closely resembles animal models of transient focal ischaemia, wherein intra-ischaemic hypothermia was more effective than delayed post-ischaemic cooling and was more effective in transient ischaemia and reperfusion models [17-23]. The IHAST trial included 1,001 good grade (WFNS grades I to III) subarachnoid haemorrhage (SAH) patients undergoing aneurysm surgery and randomised them to intraoperative normothermia (36.5°C) versus mild intraoperative hypothermia (33°C). Surprisingly, intraoperative hypothermia did not improve neurologic outcome [24]. Theoretically, the group randomised to mild hypothermia should have enjoyed the cerebroprotective effects of mild intra-ischaemic hypothermia intraoperatively, especially during the ischaemic challenge of temporary clipping. It was subsequently speculated that possibly not all IHAST patients had experienced a significant intraoperative ischaemic challenge and in turn a subgroup analysis of those 441 patients who indeed required temporary clipping was performed [25]. Among these patients, the duration of focal ischaemia - that is, temporary clipping - was the most important determinant of outcome, with those patients requiring temporary clipping of >20 minutes faring significantly worse. Even in this selected subpopulation, mild intra-ischaemic hypothermia did not significantly alter neurologic outcome after intraoperative focal ischaemia. The IHAST results raise the question of how to document relevant intraoperative focal ischaemic challenges and the beneficial effects of neuroprotective interventions in future studies. Even after SAH, gentle surgical manipulation and brief temporary clipping in expert hands were apparently not adequate ischaemic challenges to resemble animal models of temporary focal cerebral ischaemia and reperfusion.
